# Innate Structure of DNA Foci Restricts the Mixing of DNA from Different Chromosome Territories

**DOI:** 10.1371/journal.pone.0027527

**Published:** 2011-12-21

**Authors:** Pedro Olivares-Chauvet, Dorota Fennessy, Dean A. Jackson, Apolinar Maya-Mendoza

**Affiliations:** Faculty of Life Sciences, University of Manchester, Manchester, United Kingdom; Brunel University, United Kingdom

## Abstract

The distribution of chromatin within the mammalian nucleus is constrained by its organization into chromosome territories (CTs). However, recent studies have suggested that promiscuous intra- and inter-chromosomal interactions play fundamental roles in regulating chromatin function and so might define the spatial integrity of CTs. In order to test the extent of DNA mixing between CTs, DNA foci of individual CTs were labeled in living cells following incorporation of Alexa-488 and Cy-3 conjugated replication precursor analogues during consecutive cell cycles. Uniquely labeled chromatin domains, resolved following random mitotic segregation, were visualized as discrete structures with defined borders. At the level of resolution analysed, evidence for mixing of chromatin from adjacent domains was only apparent within the surface volumes where neighboring CTs touched. However, while less than 1% of the nuclear volume represented domains of inter-chromosomal mixing, the dynamic plasticity of DNA foci within individual CTs allows continual transformation of CT structure so that different domains of chromatin mixing evolve over time. Notably, chromatin mixing at the boundaries of adjacent CTs had little impact on the innate structural properties of DNA foci. However, when TSA was used to alter the extent of histone acetylation changes in chromatin correlated with increased chromatin mixing. We propose that DNA foci maintain a structural integrity that restricts widespread mixing of DNA and discuss how the potential to dynamically remodel genome organization might alter during cell differentiation.

## Introduction

Within the nucleus of higher eukaryotic cells [1]–[3] individual chromosomes are folded to occupy spatially discrete chromosome territories (CTs) (reviewed in [4]–[6]). DNA foci, which typically contain 250–1,000 kbp of DNA, provide the fundamental subunits of higher order chromatin folding within CTs. Though the molecular mechanisms that define the structure of foci are unclear, it has been known for many years that discrete foci are stable entities over many cell generations and that they contain multiple units of DNA synthesis, which are replicated together at specific times of S phase [7], [8]. This temporal regulation of replication, within defined cohorts of DNA foci, emphasises the importance of links between chromosome structure and function, while preserving epigenetic information during cell proliferation [9], [10].

As stable structures of higher-order chromatin folding, DNA foci might be expected to suppress DNA mixing [11], [12]. In fact, the dynamic mobility of chromatin within mammalian CTs is generally constrained at less that 1 µm and once nuclei are formed, following mitosis, the relative spatial distribution of CTs is largely preserved [4], [5]. The structure of individual CTs is however plastic [13], [14], so that chromatin within individual territories might assume a variety of alternative configurations [15]. Extreme examples of alternative patterns of chromatin folding are most evident in gene-rich chromosomal domains - such as the human MHC locus - which are able to form extended chromatin loops that spread away from the linked CT when gene expression is induced [16]. However, dynamic analysis of defined endogenous loci has not been possible and, as a result, large artificially-tagged ectopic repeats have been used to analyze chromatin mobility in mammalian cells [17].

Over the past few years an alternative view of chromosome structure has emerged, which challenges the idea that CTs are self-contained and proposes that significant mixing of DNA can occur [2], [18]. Clear evidence for long-range chromatin looping evolved from the analysis of intra-chromosomal interactions during gene expression, using chromosome conformation capture (3C) technologies. More surprisingly, while evaluating the extent of the regulatory interaction it became clear that genes from different CTs were also able to co-associate at common sites of gene expression [19], [20]. However, validation of specific inter-chromosomal interactions within individual cells typically demonstrated that only ∼10% of the loci in question were co-associated when transcribed [19], [21], [22]. Nevertheless, recent innovations in analysis of genome-wide interaction networks or functional ‘interactomes’, have placed unprecedented emphasis on understanding how chromatin dynamics facilitate the formation of gene interactions networks, which in turn might contribute to the regulation of gene expression in mammalian cells [18], [23].

If long-range chromosomal interactions make a significant contribution to the regulation of gene expression in higher eukaryotes, it is important to understand the range and extent of interactions that this involves. To address this issue, we have used single cell imaging techniques to monitor chromatin mixing in human HeLa cells. DNA foci were pulse-labeled using fluorescent dNTP analogues that incorporate during replication and remain stably associated with labeled CTs for at least 14 days. After labeling, mitotic segregation reveals discrete chromatin domains with clearly defined DNA foci, so that the dynamic properties of foci and interactions between foci of neighboring CTs can be assessed. We show that while individual foci are spatially dynamic their DNA is generally locally constrained and so limits mixing between neighboring CTs.

## Results

### Chromosome territories are discrete structures

**Figure 1:**
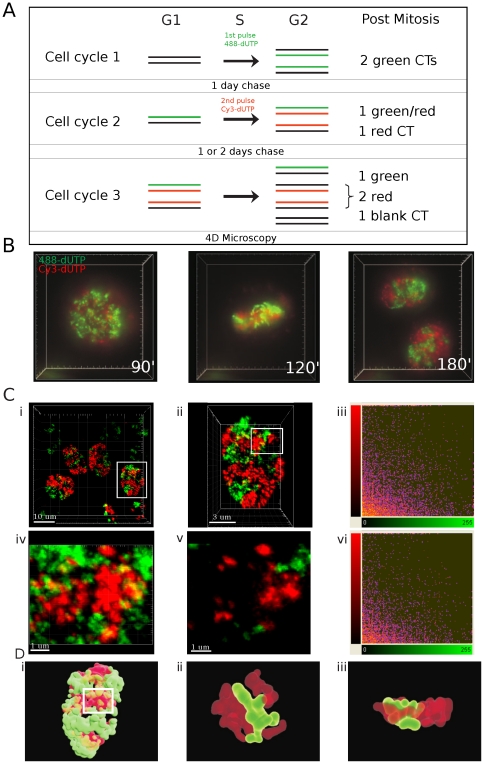
DNA foci are discrete higher-order chromatin structures. DNA foci within HeLa cells were labelled in two consecutive cell cycles - using AF488-dUTP in the first cycle and Cy3-dUTP in the second – and grown for a further 1–2 days to resolve the labeled DNA foci into uniquely labeled nuclear domains (A). In this context, the domains represent clusters of CTs as individual CTs cannot be resolved with confidence. Labeled cells were analyzed using 3-D time-lapse microscopy (DeltaVision) for up to 24 h (B; time-lapse frames are taken from Supplementary video S1), to confirm that chromosome in mitosis are labeled with either the red or green fluorescent precursor. Cells like those shown (B) were also fixed and imaged (Zeiss LSM510META) without further processing (C). For image analysis, cells with similar intensities in the two imaging channels were manually selected (Ci - white box), confocal Z stacks collected and 3-D projections generated (Cii). Nuclei like that shown in Cii were used for co-localization analysis, using all voxels within the image (Ciii). For this example, co-localization analysis using Imaris software (Ciii), gave a Pearson's coefficient (−0.0194) consistent with very weak co-localization. The region highlighted in Cii (white box) contained the majority of voxels containing signal from the red and green channels and was selected for further analysis. A high magnification view of the cropped region shows the local structure of chromatin domains, both in the entire Z series (Civ) or individual sections (Cv), with discrete patches of red and green labeling and little mixing (yellow) of signal from the two channels. Co-localization analysis using Imaris software without background adjustment (Cvi) showed this typical sample to have a Pearson's coefficient of −0.0437. A surface rendered video simulation of chromatin domains in the cell from Cii was also generated to show the distribution of interaction interfaces within the sample (D; see Supplementary video S2 to section though the 3-D reconstruction). High-power views of the 3-D region highlighted (white box in Di) are shown (Dii–iii). This modeling demonstrates the complex structure of interaction surfaces, with many surface protrusions from one chromatin domain interdigitating with folds or channels within the neighboring domain. Two snap-shots from the 3-D reconstruction (Supplementary video S3) are shown. Scale bars of 10, 3 or 1 µm are shown on individual panels.

We tested the extent of DNA mixing between CTs using established protocols that allow CTs and individual DNA foci to be visualized in living cells [24], [25]. Cells were pulse-labeled with AF488-dUTP, grown for 24 h and then pulse-labeled with Cy3-dUTP and grown for a further 1–2 days (Fig. 1). Because replication is semi-conservative and mitotic chromosome segregation is random, this protocol yields cells that contain uniquely red or green labeled CTs together with a minority of CTs that are unlabeled (Fig. 1A).

Live cell analysis (Fig. 1B and video S1) showed that the identity of CTs is preserved for many hours with little or no interaction between neighboring CTs. However, as resolution is limited by the low levels of illumination used during live cell imaging we also performed imaging on fixed cells (Fig. 1C). Post-fixation analysis, in the absence of processing that might perturb chromosome structure at the resolution analyzed by light microscopy, allows the structure of the differentially labeled chromatin domains and distribution of their foci to be visualized (Fig. 1Ci; projections of complete Z stacks are shown). With this type of analysis, the structure of DNA foci is clearly preserved and foci are clustered into local domains that represent individual or small groups of CTs. Notably, the boundaries between adjacent green and red domains are clearly defined (see isolated channels in Fig. S1) and regions of apparent co-localization between the differentially labeled regions (yellow regions; high magnification views in Fig. 1Civ–v) were restricted to these boundary domains. However, rotation of the 3D image suggested that many sites of apparent localization resulted from the spatial overlap of adjacent foci in projections of optical sections and not true co-localization within individual voxels of the 3D image (Fig. 1D and videos S2 and S3). To address this issue, we next attempted to place numerical limits on the low-level co-localization seen by measuring both the nuclear volume occupied by the co-localized regions and the amount of labeled DNA within these domains of chromatin mixing.

### Quantitative measurement of inter-chromosomal mixing

A number of strategies have been described for monitoring levels of co-localization in confocal images (reviewed in refs [27], [28]). Pearson's and Mander's coefficients provide a qualitative insight into degrees of co-localization on double stained confocal images. The Mander's coefficient is scaled from 0 to 1, where a value of 1 represents complete co-localization and a value of 0 no overlap between the imaging channels. For the Pearson's coefficient (PC) the scale is 1 to −1. On this scale, 1 represents complete co-localization and negative numbers represent exclusion, with −1 representing samples with no overlap at all.

While Pearson's and Mander's coefficients are used routinely for analysis of signal co-localization it is important to recognise that these values are heavily influenced by the way in which noise and background labeling in the sample is treated. Quantitative image processing is notoriously challenging, principally because of uncertainties in setting threshold values that reliably define true signal from various sources of noise [26]. However, the labeling protocol applied here avoids traditional sources of background staining (such as those that arise during immuno-labeling), as the fluorescent replication precursor analogues are incorporated directly into DNA. By the time imaging is performed, essentially all fluorescent molecules added to cells are covalently bound to DNA and make no contribution to background.

Another challenge of image analysis is that raw images often contain electronic noise, which is characterized by isolated voxels with high signal intensities. Imaging software provides different strategies to remove such noise. Gaussian filtering averages the signal in neighboring voxels and so smoothes noise and allows extraneous signal to be subsequently removed by thresholding. For quantitative analysis, however, one limitation of this approach is the incorporation of unreal voxel intensity values into the data set, which tend to degrade the signal and compromise the integrity of structures by spreading of their edges. Median filtering provides an alternative strategy in which each voxel in the image is assigned the median value of all of the immediately adjacent voxels. Hence, isolated high-intensity voxels will be eliminated, while voxels storing real signal are essential unaltered by the filtering step.

After labeling (Fig. 1A), maximum projections of complete Z series were collected (Fig. 1Ci; taken using a 100× lens). Individual nuclei with classical patterns seen during early S phase were then selected for detailed analysis based on the balance of labeling in the red and green channels (Fig. 1Cii shows an electronic zoom of the selected cell from Fig. 1Ci). Prior to image analysis, low level electronic noise in the zoomed image was extracted using a median filter (3×3×3 voxels). We then applied an empirical approach for manual background adjustment and found that within our samples the best estimate of background was represented by a signal corresponding to the standard deviation of the average signal intensity across all labeled voxels in the image (Fig. S1). Ten nuclei like the typical sample shown (Fig. 1Cii) were then analyzed to monitor levels of channel co-localization (Table 1). Across this sample, all approaches yielded a negative average Pearson's coefficient and low Mander's coefficient, consistent with very low levels of co-localization between the two imaging channels.

**Table 1:** Analysis of different approaches for signal co-localization.

Summary10 nuclei	No threshold			Thresholded		
Coloc. Metric	PC	Mander's		PC	Mander's	
		Green	Red		Green	Red
Raw files (no filtering)	−0.03646	0.18368	0.05448	−0.05216	0.09689	0.0401
Median Filter 3×3×3	−0.07823	0.11228	0.01549	−0.10285	0.04953	0.01616
Gaussian Filter 0.08 µm	−0.04365	0.5957	0.42345	−0.11055	0.1041	0.07763

A variety of automatic and manual protocols were tested to monitor levels of co-localization in samples generated throughout this study. Confocal series were collected (with sequential imaging in the labeling channels) and data files imported into image analysis software (Imaris suite). Pearson's and Mander's coefficients were used as indicators of the extent of co-localization between different channels (see text). Entire confocal series for 10 different nuclei (like those shown in Fig. 1C) were used to analyze apparent co-localization between the imaging channels using the different conditions identified in the Table, as discussed in the text. It is notably that the different conditions used have only a superficial impact of levels of co-localization, with very weak co-localization seen in all cases. Simple median filtering improves the quality of the images and decreases apparent co-localization relative to the unprocessed images. However, using Gaussian filtering as an alternative dramatically increases the apparent co-localization, by spreading the edges of the labeled structures (this is evident from differences in the respective Mander's coefficients). Thresholding after preliminary processing (filtering) eliminates low-intensity volexs but reduces levels of co-localization only slightly.

We also performed co-localization analysis after selecting regions of interest to exclude the contribution of black voxels that lie outside the nucleus (Fig. 1Civ–vi). Importantly, for this analysis we selected nuclear regions with the highest levels of apparent co-localization with adjacent red and green chromatin domains (Fig. 1Civ). Within these regions, the average Pearson's coefficient within such cropped regions (n = 10; vol = 28.5 µm^3^) was −0.07 ±0.04 and the Mander's coefficient was 0.05 ±0.04 and 0.08 ±0.06 in the green and red channels, respectively. Within the selected regions of interest, the average co-localized volume covered 0.28±0.24 µm^3^ occupying 0.96%±0.85 of the cropped box. The green signal occupied on average 17.3%±7 of the imaging voxels and the red signal 11.4%±3, so the labeled space represents ∼30% of the volume in these selected regions. By analyzing the most highly intermingled chromatin domains within individual nuclei, this analysis provides an upper limit for the proportion of the total nuclear volume in which inter-chromosomal mixing of the labeled chromatin is seen.

**Figure 2:**
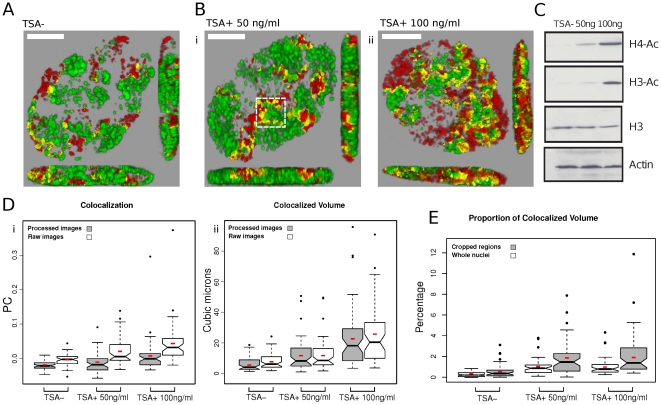
Chromatin epi-states define foci structure. AF488- and Cy3-dUTP were incorporated into DNA foci are described in the legend to Figure 1. Prior to imaging, cells were treated with TSA (24 h) at the concentrations shown (A–E). For imaging, samples were fixed and confocal Z series collected (Zeiss LSM710) and processed (A–B); imaging was performed on double-labeled nuclei but without selection for labeling intensity. Changes seen in the structure of DNA foci following treatment with TSA (Bi–ii) correlated with changes in global histone acetylation by Western Blot analysis using specific antibodies to pan-Ac+ histones H3 and H4 (C). Image processing of the confocal projections (n = 50 per sample) was performed using Fiji and jacop software. Analysis was perform on raw images, without processing, and on the same images after processing as describe in the legend to Figure 1 (D). As seen in Figure 1, untreated cells (A) gave a negative Pearson's coefficent (Di) consistent with low levels of colocalization in the sample. Following TSA treatment (B), a significant increase in Pearson's coefficient was recorded, demonstrating increased co-localization (Di). In order to develop quantitative estimate of channel co-localization, voxel-level channel intensities were extracted and the volume (µm^3^) of co-localized voxels calculated (Dii). Finally, the co-localized volumes were calculated as a proportion (%) of the total nuclear volume (whole nuclei) and the volume of the most highly labeled regions (cropped regions – the boxed region in Bi shows a typical example) of individual nuclei (E; Table 2). Small red boxes on the box plots represent the mean value for each distribution. Scale bars of 5 µm are shown on individual panels.

Co-localization analysis is most reliable when the two imaging channels are labeled with similar intensities and signal fills the full dynamic range of the detectors used. Hence, for the preliminary analysis shown (Fig. 1 and Table 1) co-localization analysis was performed on manually selected images with similar intensities and label distribution in the two imaging channels. However, as image selection might bias analysis, we next analyzed larger data sets without prior sample selection (Fig. 2A).

### The local chromatin environment defines the integrity of DNA foci

As part of a detailed analysis of the structure of DNA foci in untreated cells, we also evaluated if the integrity of foci was influenced by the local chromatin environment. Molecular mechanisms that define the higher order structure of chromatin domains are unknown. However, as foci within the euchromatin and heterochromatin compartments – which are labeled at defined times of S phase – persist over many cell generations it is reasonable to suggest that the chromatin environment contributes to the preservation of these structures. To evaluate if the epigenetic status of chromatin influences the structure of DNA foci, we analyzed foci in cells treated with the histone deacetylase (HDAC) inhibitor TSA [29]. As before, replicating DNA was labeled using double-pulse strategies and individual CTs resolved through random mitotic segregation during cell proliferation. Cells with discrete foci were then treated with TSA and imaging performed 24 h later (Fig. 2).

As discussed above, for detailed quantitative analysis, double-labeled cells were randomly selected and 3D images stack generated (Fig. 2A–B); as before, only nuclei with labeled early S phase foci (i.e. euchromatic) were used for subsequent analysis. Cell populations were processed either as raw images or after filtering and thresholding as described (Fig. S1) and statistical tests performed (not shown) to establish that the analysis of 50 cells/sample was sufficient to ensure reliability of the data. In parallel samples, cells were treated using 2 concentrations of TSA (Fig. 2), which were selected based on the extent of changes in acetylation of histones H3 and H4 (Fig. 2C). Untreated (control) samples contained discrete DNA foci that were distributed in distinct domains with regions of co-localization restricted to the boundaries of adjacent domains. In this data set, cropped regions with the highest levels of co-localization contained only 0.55+/−0.6% of co-localized voxels when, on average, 27.8% of voxels in the selected regions were labeled (Table 2).

**Table 2:** Comparison between co-localization results for entire nuclei and cropped regions.

	Vol Coloc. (µm^3^)	% Coloc.	% Green	% Red	PC	M Green	M Red	%occupied
**Nuceli n = 50**								
**TSA−**	6.03±4.49	0.30±0.22	8.48±2	4.73±1.7	−0.020±0.01	0.03±0.02	0.05±0.02	13.21
**TSA+ 50**	12.23±10	1.01±0.8	12.06±4.2	7.71±2.5	−0.015±0.02	0.07±0.04	0.11±0.07	19.77
**TSA+ 100**	23.90±19	0.98±0.8	6.86±2.33	9.75±2.7	0.006±0.05	0.12±0.08	0.09±0.07	16.61
**ROIs n = 50**								
**TSA**−	0.36±0.4	0.55±0.6	13.87±4.3	13.93±5.6	−0.099±0.03	0.03±0.03	0.04±0.03	27.81
**TSA+ 50**	1 ±0.6	1.8±1.6	19±8.3	19±5.7	−0.13±0.07	0.08±0.06	0.09±0.06	39
**TSA+ 100**	1.55±2.7	2.15±2.07	17.50±4.5	21.97±5.6	−0.1087±0.08	0.12±0.1	0.1±0.1	40

Data from experiments described in Figure 2 are shown. Cells were labeled and processed as described and 50 whole nuclei of selected cropped regions (ROIs) from each nucleus were analyzed. Cropped region of the same surface area were selected to contain the most highly co-localized nuclear region. A typical example is highlighted in Figure 2Bi (boxed area) in which a regions of substantial chromatin mixing (yellow) lies at the junction of discrete red and green chromatin domains. The total volumes of nuclei and selected cropped regions were 1953±946 µm^3^ (n = 150) and 65±7 µm^3^ (n = 150), respectively. No statistically significant differences in the nuclear volume of TSA treated cells were seen.

When cells were treated with TSA a clear increase in channel co-localization was seen (Fig. 2 and Table 2). When Pearson's correlation coefficient was used as an indicator of co-localization, differences were statistically significant when cells were treated with TSA at 100 ng/ml and an intermediate level of co-localization was seen when 50 ng/ml was used (Fig. 2D and Table 3). Importantly, the same trends were seen when analysis was performed on raw images, without processing, or after median filtering and thresholding (Fig. 2Di). However, as Pearson's correlation coefficient provides an abstract indicator of channel cross-talk or co-localization, we also deconstructed images and used a volex level co-localization analysis to calculate the volume of voxels that contained both labels (Fig. 2Dii and E). This analysis confirmed that the co-localized volume in the nuclei of untreated control cells was restricted to ∼6 µm^3^, representing 0.3% of the nuclear volume, whereas following treatment with TSA at 100 ng/ml the co-localized volume increased to ∼24 µm^3^ (Table 2).

**Table 3:** Statistical Analysis of co-localization results.

Pair-wise Mann-Whitney		
Nuclei		
	TSA+50 ng/ml	TSA+100 ng/ml
**Pearson's Coefficient**		
TSA−	6.2E-01	5.5E-06
TSA+ 50 ng/ml	x	1.83E-03
**Co-localized Volume**		
TSA−	2.482E-04	9.457E-12
TSA+ 50 ng/ml	x	8.154-05
**Co-localized Percentage**		
TSA−	7.616E-10	4.923E-11
TSA+ 50 ng/ml	x	8.931E-01

ROIs		
	TSA+50 ng/ml	TSA+100 ng/ml
**Pearson's Coefficient**		
TSA−	5.88E-03	3.519E-02
TSA+ 50 ng/ml	x	4.645E-01
**Co-localized Volume**		
TSA−	3.705E-08	5.922E-11
TSA+ 50 ng/ml	x	1.564E-01
**Co-localized Percentage**		
TSA−	8.593E-09	1.544E-10
TSA+ 50 ng/ml	x	6.951E-01

Kruskal-Wallis test		
Nuclei		ROIs
**Pearson's Coefficient**		
3.88E-05		1.481E-02
**Co-localized Volume**		
1.166E-11		1.13E-11
**Co-localized percentage**		
1.68E-12		1.513E-11

As the distributions of the values from the three different treatments are not normal, non-parametric methods were used. The Mann-Whitney test was used for pair-wise tests and the Kruskal-Wallis test for multiple comparisons.

**Figure 3:**
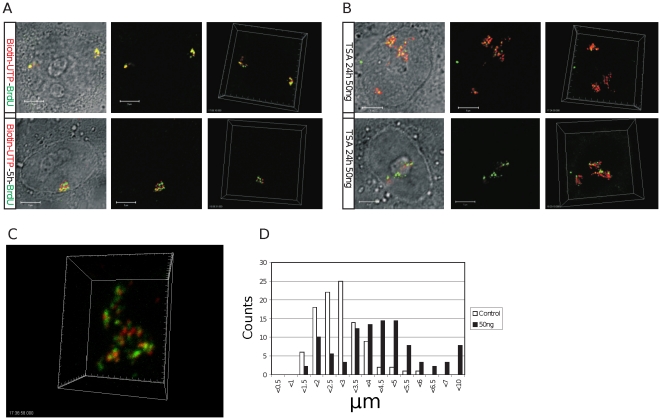
The chromatin environment contributes to the local and long-range architecture of DNA foci and CTs. HeLa cells were pulse-labeled with biotin-dUTP and BrdU either sequentially or with an unlabeled intervening chase of 5 h and then grown for 6–7 days to resolve individual CTs by random mitotic segregation. Cultures were then divided into 2 and treated without (A) or with (B–C) 50 ng/ml of TSA for 24 h. Simple visual inspection showed CTs to be visibly disorganized and expanded (C – shows an isolated CT from the sample in B) with notable deterioration in the structure of DNA foci, which appeared irregular and diffuse relative to untreated controls. Expansion of CTs was confirmed by measuring the diameter (long axis) of individual territories (D). Though the structural changes were obvious in pulse-labeled samples (compare A and B) for this analysis we used cells that were labeled with BrdU throughout S phase so that the boundaries of individual CTs could be identified with confidence. The diameter of CTs (D) within control cells (open bars; dia = 2.65 µm +/−1.25; n = 100) was seen to increase by 1.59-fold in TSA treated cells (closed bars; dia = 4.21 µm +/−1.68; n = 90; t test −p<1.3×10–12). Under these conditions, there was no significant change in the average nuclear volume of the two samples. Scale bars of 5 µm are shown on individual panels.

Many experiments support the idea that euchromatic and heterochromatic DNA foci have distinct characteristics that contribute to the spatial organization of CTs [4]–[6]. To assess how these specialized chromatin states contribute to CT structure, we analyzed DNA foci within isolated CTs that were labeled with biotin-dUTP (early S phase) and BrdU (mid/late-S phase) using a pulse-chase-pulse strategy (Fig. 3). After labeling, cells were grown for 5 days to reveal isolated CTs, treated with TSA for 24 h and the structure of DNA foci and CTs analyzed. In comparison with untreated control cells from the same labeled population (Fig. 3A), the DNA foci of cells treated with TSA were clearly swollen and dispersed (Fig. 3B–C), consistent with the local mixing of adjacent foci seen along the boundaries of neighboring CT (Fig. 2B). However, despite the clear structural deterioration and associated >2-fold increase in CT volume (Fig. 3D) widespread mixing of the early and late chromatin domains was not seen (Fig. 3), suggesting that even following TSA treatment some residual higher-order structure is preserved. Based on these observations, we propose that the chromatin environment has a significant influence on the structure of DNA foci and that patterns of interaction between foci contribute to the spatial architecture of CTs.

### Analysis of completely labeled CTs

Fluorescently labeled replication precursor analogues have distinct technical advantages for image analysis (Figs. 1 and 2) but are limited by the extent of incorporation achieved; under conditions used here, which do not compromise the rate of DNA synthesis, the modified precursors are only incorporated into growing replication forks for ∼15 min before the labeled precursor dNTPs are consumed. This limitation means that alternative strategies must be used to provide global estimates of DNA mixing within mammalian nuclei.

**Figure 4:**
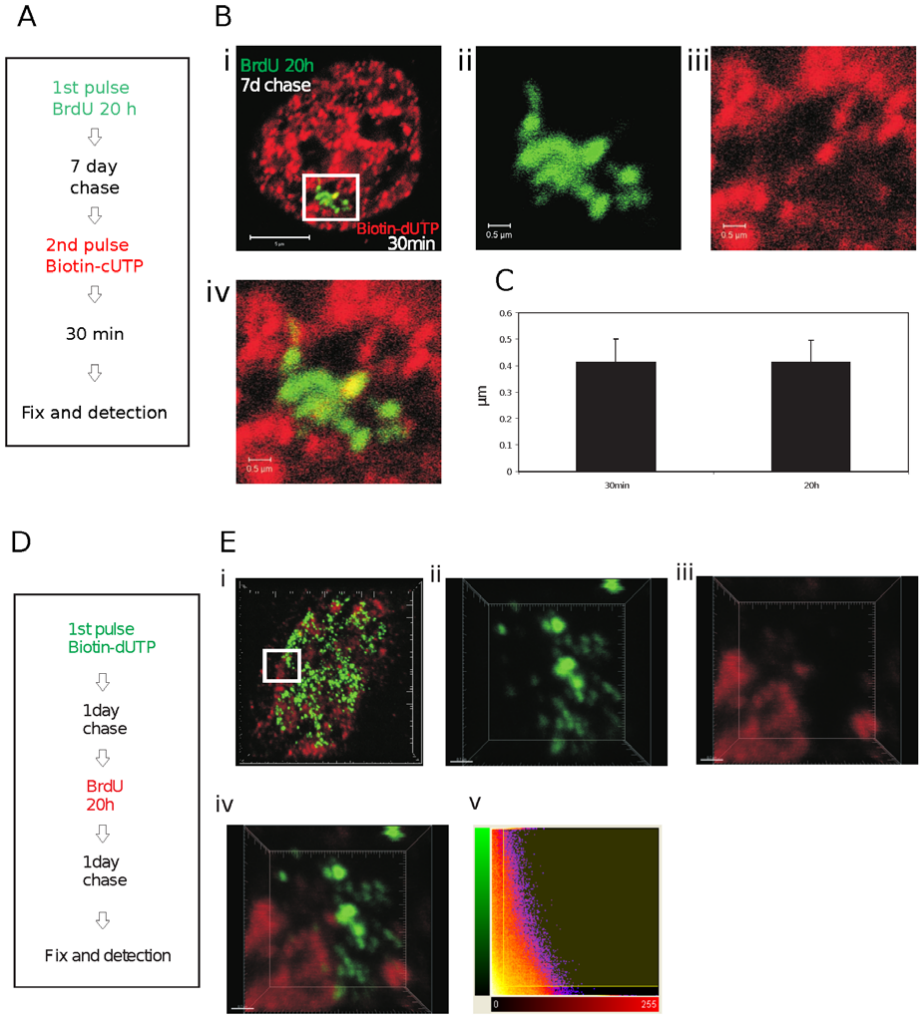
Estimating global levels of chromatin mixing. Pulse-labeling with conjugated replication precursors such as Cy3- or biotin-dUTP yields labeled DNA foci in which only about 15% of DNA contains the modified precursor. As we are able to measure DNA mixing at the boundaries of such foci in highly labeled nuclear volumes, it is important to know if apparent volumes of foci are influenced by the extent of modified precursor incorporation. HeLa cells were labeled as shown (A) and processed by indirect immuno-labeling. Confocal projection of double-labeled cells like that shown (B) were collected and the diameters of foci measured (C) using Imaris software in selected regions as shown (a zoom of the boxed region in Bi is shown as individual channels in Bii and Biii and Biv shown the channel merge). Foci that were pulse-labeled with biotin-dUTP had an average diameter of 0.413+/−0.087 µm (mean+/−SD; n = 100). Foci that were labeled for the entire S phase with BrdU had an average diameter of 0.414+/−0.081 µm (mean+/−SD; n = 100). Bars are 5 and 0.5 µm in low and high power images, respectively. To increase the extent of labeling in one imaging channel, foci within individual CTs were labeled with either BrdU or biotin-dUTP using the labeling scheme shown (D). Samples were fixed and processed to visualize site of incorporation by indirect immuno-labeling using secondary antibodies conjugated with Qdots™; Qdots are very stable during illumination and allowed sampling using 50 nm Z steps (92 slices in the examples shown) and multiple scans without bleaching. Individual cells were selected and confocal projections generated (E). Regions from selected cells were analyzed to identify the extent of co-localization between the two labeling channels. A 3-D reconstruction of the region highlighted in (Ei) was used for further analysis (Eii–Ev: Eii (green) and Eiii (red) show signal in the separate labeling channels; Eiv an overlay of the red and green channels and Ev a co-localization analysis using Imaris software). Note the discrete nature of the labeled sites in both labeling channels and almost complete lack of sites of overlap (yellow) in the channel merge (Eiv) – in this typical example the co-localized volume was 0.96%. Scale bars are 5 and 0.7 µm in low and high power images, respectively.

We considered two strategies for estimating genome-wide levels of inter-chromosomal DNA mixing. First, because euchromatin and heterochromatin occupy discrete nuclear domains the distribution of these chromatin compartments is non-uniform [4]–[6]. Hence, even in nuclei with partially labeled genomes it should be possible to estimate the maximum extent of DNA mixing locally using volumes of the nucleus in which the majority of DNA foci are labeled. This approach was tested above (Figs. 1 and 2) using crops of the most highly labeled nuclear regions. Within these highly labeled domains, typically ∼30% of the cropped nuclear volume was occupied by labeled DNA. Moreover, as 50% or less of the nuclear volume is occupied by chromatin [30], we can estimate that >60% of the chromatin space present in the selected regions will be labeled. Given the short duration of labeling with modified dNTPs this might seem surprising. However, when the structure of DNA foci that had been pulse-labeled with biotin-dUTP were compared with foci labeled with BrdU throughout S phase no significant differences were seen (Fig. 4). This shows that when the DNA within individual foci is only partially labeled the plasticity of chromatin folding means that the labeled and adjacent unlabeled regions cannot be resolved. In fact, as typical foci have 3–5 replicons and so 6–10 extending replication forks, it is not surprising that the labeled and unlabeled regions cannot be resolved by confocal microscopy.

For the second approach we analyzed cells with DNA that had been labeled with BrdU throughout S phase. Prior to this analysis we performed an extensive analysis of labeling specificity [31], and found that DNA foci labeled with BrdU or biotin-dUTP could be distinguished with good sensitivity and without cross-talk between the imaging channels. A pulse-chase-pulse-chase labeling strategy (Fig. 4D) was used to monitor the interaction between DNA foci of neighboring Br and biotin-containing nuclear domains (Fig. 4E). As before (Figs. 1 and 2) regions of co-localization were clearly restricted to boundaries between these neighboring domains. In the cropped region highlighted (Fig. 4Ei), the Pearson's coefficient is −0.3036, consistent with exclusion of signal in the two labeling channels (Fig. 4Ev). As in this typical example, even when individual CTs are completely labeled with BrdU, we see that only ∼1% of the nuclear volume contains both Br and biotin-labeled DNA.

### Chromatin dynamics in living cells

In seminal studies on CT dynamics, Cy3-labeled DNA foci in HeLa and SH-EP N14 (a neuroblastoma cell line) cells were shown to undergo constrained random diffusion with rare examples of directional motion correlating with changes in cell shape [13]. Later, a more sophisticated analysis of temporal dynamics was performed using a ∼10 Mbp artificial repeat that was able to bind lacI-GFP [17], [32]. However, largely because of technical limitations, we have only limited understanding of dynamic changes that occur when DNA foci engage DNA or RNA polymerases to function as a synthetic template [13], [14], [32]–[34].

**Figure 5:**
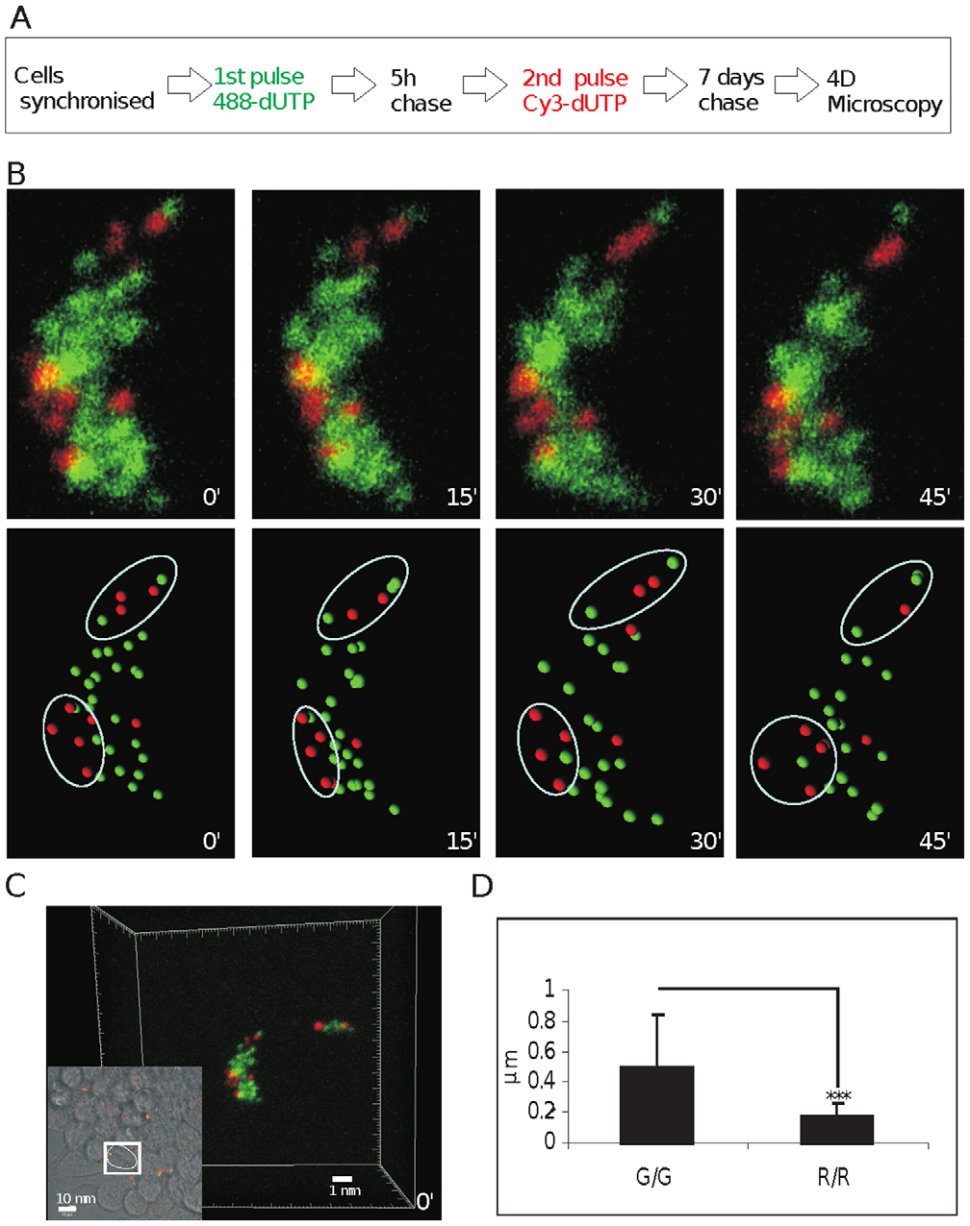
Differential dynamic behavior of DNA foci labeled during early and mid/late S phase. Early replicating euchromatic foci were pulse-labeled with AF488-dUTP (green) and mid/late replicating foci with Cy3-dUTP (red) - an optimal pulse separation of 5 h was established experimentally (A). Individual CTs were resolved by mitotic segregation for 7 days (A–C) and confocal time-lapse microscopy (Zeiss LSM510META) performed over 1–2 h with sampling every 15 min. Raw images (B; upper panels) show maximum projections of Z stacks for a typical isolated CT (from the cell highlighted in C). Raw images were imported into Imaris software in order to determine the mass centers of individual labeled sites (DNA foci). Software-generated spheres (250 nm; B; lower panels), which represent the mass centers of discrete foci, were used to develop 3-D coordinates to define changes in separation of paired neighboring foci (n = 42). For each time-lapse series used (B), 4 3-D projections were generated at 15 min intervals and specific regions identified (white ovoids) where foci could be tracked without ambiguity. In each image, the separation between all neighboring pairs of assigned foci (within each ovoid) was determined. Finally, the 4 data sets were used to calculate 3 relative separations that define the change in separation of assigned foci in µm/15 min (µm in D). The relative dynamic properties shown relate to early foci within early replicating chromosomal domains (G/G) and mid/late foci within mid/late replicating domains (R/R). Scale bars of 1 and 10 µm are shown on individual panels.

As structural transitions related to chromatin function must increase the probability of DNA mixing within the inter-chromatin domain, we next wanted to evaluate if live cell imaging could be used to define the structural stability of DNA foci. Because DNA foci within euchromatin and heterochromatin have well-characterized nuclear organization [15] and dynamic properties [14], it is possible to use foci labeled at different times of S phase as meta-stable landmarks to map the relative movement of individual foci. A typical example of this approach is shown in Figure 5. Replication foci were labeled with AF488-dUTP during early S phase and Cy3-dUTP 5 hours later (Fig. 5A). Using this labeling program, the relative spatial stability of heterochromatic foci labeled during mid-S phase (red) can be used as anchor points to align CTs at different times during the imaging series and so increase the confidence with which the location of individual foci can be assigned. In the example shown, the overall shape of the CTs and local architecture of individual foci is maintained throughout the imaging time-course even though it is not unusual to see local transformations in the shape of individual CTs; the example shown here is seen to rotate around its vertical axis (Fig. 5B).

Even though the quality of foci is limited by the imaging set-up (low laser power) used during live cell imaging, the type of data shown in Figure 5 allows unambiguous identification of discrete foci using image processing software (Fig. 5B). In this typical example, individual foci are most obvious along the periphery of CTs (Fig. 5B - regions highlighted in white ovoids). In such areas of the sample, the ability to track and assign co-ordinates for individual foci during time-lapse imaging allows the location and movement of individual foci to be monitored with confidence. As noted before [13], [14], we found euchromatic foci to be locally dynamic, typically moving ∼0.5 µm over periods of 15 minutes (Fig. 5D). However, dramatic directional movements were never sustained for long periods. Instead, foci appeared to oscillate within CTs so that individual territories maintained their relative position and general shape for many hours. Relative to euchromatic foci, heterochromatic foci were frequently clustered and showed significantly reduced mobility (Fig. 5D). This correlates with heterochromatic foci being preserved as temporally stable clusters of structurally inert chromatin. The architecture of mid/late replicating DNA foci correlates with the structural polarization of CTs and corresponding programme of DNA synthesis in mammalian cells [35].

## Discussion

Competing models of nuclear organization have addressed how prevailing views of CT structure and chromatin dynamics might be resolved [36]–[38]. Traditionally, fluorescent in situ hybridization (FISH) has been used to define the distribution of DNA from individual chromosomes. This ‘chromosome painting’ of intact nuclei showed CTs to be discrete structures [4]. However, quantitative analysis of low-level surface mixing is technically challenging within the 3D volume of an entire CT. To address this point, Branco and Pombo [39] applied routine FISH techniques to ∼200 nm cryosections. With this approach, the borders of neighboring CTs were seen to contain extensive domains of inter-chromosomal mixing. For example, when PHA stimulated human lymphocytes were analyzed, ∼40% of the chromatin-rich compartment – corresponding to ∼20% of the nuclear volume - was estimated to contain DNA from more than one chromosome.

In higher eukaryotes, we have limited information about the range and scale of chromatin dynamics and the potential for inter-chromosomal mixing in living cells. Individual CTs within mammalian nuclei are known to be locally plastic [13], [15] and many have structures that change both locally [14] and at longer range [16], [40] in response to changes in gene expression. However, detailed dynamic studies on specific endogenous loci have not been reported. Moreover, with live imaging, it is extremely difficult to reliably measure subtle changes in shape and intensity of 3D structures based on fluorescent time-lapse imaging. Hence, it is unclear how the structure of DNA foci changes when functions such as DNA or RNA synthesis are performed (e.g. [13], [14]).

The molecular mechanisms that define the structural properties of DNA foci have not been explored in detail. It is known that individual foci within euchromatin and heterochromatin are discrete entities, implying that the local chromatin environment contributes to their structure [12], [15]. It is not clear if individual foci have strictly defined boundaries or how possible boundaries might be formed. Even so, a recent study using an unbiased genome-wide 3C approach – termed Hi-C - has demonstrated that the analysis of cell populations shows DNA to be clustered into ∼1,000 kbp chromatin domains [41]. The DNA domains that were predicted during analysis of the Hi-C data show a strong size correlation with DNA foci [8] and replication timing domains [42]. Interestingly, bioinformatic analysis has predicted that at least part of the structural organization of the higher-order chromatin domains or globules correlates with the distribution of the insulator protein CTCF on DNA [43], [44] and the association of active genes within common transcription factories [43]–[45].

### Structural plasticity of CTs and DNA foci

In this study, we used a single cell approach to evaluate if the structure and dynamic behaviour of CTs could be rationalized with the formation of wide-scale genomic interaction networks, which can be crudely defined by the extent of inter-chromosomal mixing within nuclei. We used fluorescent thymidine analogues to label DNA foci in living cells and then used light microscopy to monitor both the structure and dynamic behaviour of individual foci and CTs. Using this approach, we saw little evidence for extensive zones of DNA mixing between the foci in adjacent chromatin domains (Figs. 1, 2). Indeed, analysis of the co-localization of DNA from neighboring CTs suggests that nuclear domains in which chromatin from different CTs is freely mixed represents a small fraction – probably no more than 1% - of the chromatin space. The very limited mixing that we see agrees with other reports [11], [12] but appears at odds with the existence of widespread chromatin domains within which DNA from different chromosomes is mixed [39].

Whether this apparent discrepancy results from experimental differences or innate differences in the cell systems used is presently unclear. Our analysis is dependent on metabolic labeling during DNA replication and transformed human HeLa cells are ideally suited for this approach. Pombo and colleagues used freshly isolated peripheral human lymphocytes, which in some cases were activated by PHA treatment [39], [46]. One possibility might be that the observed differences reflect changes in higher-order chromatin architecture in transformed and specialized cells. Various technical limitations might also contribute to the differences seen using different experimental strategies. Notably, our preferred analytical strategy simply involves fixation and analysis of higher-order DNA structures that were fluorescently labeled during DNA replication (Fig. 2). Pombo et al. used technically elegant in situ hybridization to visualize the distribution of CTs. Unlike our non-destructive labeling, hybridization demands that the target DNA is denatured, which even in fixed samples might involve some loss of local structure. In addition, our metabolic labeling approach allows visualization of DNA foci [8] in samples without background (i.e. unincorporated) label. In contrast, when analysis is based on DNA hybridization, samples often contain low-level background staining, which makes true signal difficult to define [36].

### Dynamic DNA foci and chromatin looping

Our observations suggest that the chromatin in human HeLa cells does not undergo wide-scale inter-chromosomal mixing (Figs. 1, 2, 3, 4). From our analysis, we estimate that within individual cells only ∼1% of DNA is found to occupy nuclear sites where DNA from different chromosomes is likely to be freely mixed. This level of potential interaction does however reflect a snap-shot in time and it is also important to emphasize that CTs [13]–[16] and their constituent DNA foci [14], [15] are dynamic and able to engage in structural transformations (Fig. 5), so that different loci might interact with numerous other loci at different times. It is reasonable then to assume that such changes will respond to the functional state of chromatin, and not difficult to imagine how post-translational histone epi-states define a chromatin landscape, which also contributes to patterns of DNA interaction. In addition, while specific patterns of inter-chromosomal interactions might form preferred steady-state structures in differentiated cells it is important to consider how such interactions might be influenced by the formation of chromosomes and their CTs during cell division. Chromosome condensation will inevitably disrupt inter-chromosomal DNA interactions that exist during interphase and so reset the interaction networks to a structural ground-state that will be based on local structure.

While DNA foci with ∼1 Mbp of DNA are widely accepted as fundamental higher-order features of chromosome structure surprisingly little is known about the molecular principles that regulate chromatin function within these structures. Though the formation of foci is unlikely to reflect a single mechanism, it is notable that the foci which form within the euchomatin and heterochromatin compartments are distinct. This is consistent with the local chromatin environment contributing to the structure and stability of individual foci. To test this possibility, we perturbed the local chromatin environment within DNA foci by manipulating the acetylation status of histones using the histone deacetylase inhibitor TSA. After treatment with TSA, under conditions that increased global histone acetylation ∼5-fold, clear changes in the structure of DNA foci were seen (Figs. 2, 3). Notably, foci became more open or dispersed and this correlated with a 4-fold increase (Table 2) in the volume of nuclear domains where DNA from adjacent chromosomes was intermingled. TSA-induced changes in the structure of DNA foci also correlated with a more general disorganization of CTs, which showed widely variable structures and increased size (Fig. 3). These experiments show that the chromatin environment contributes to the structure of DNA foci so that when the chromatin environment is perturbed a corresponding deterioration in the structure of DNA foci and CTs is seen.

Inside the nucleus, DNA and RNA synthesis are performed within the inter-chromatin compartment, and not within the chromatin-rich DNA foci themselves [36]–[38]. Because of this spatial separation, it is self-evident that chromatin loops must be extruded from the foci towards the active sites during synthesis. This requirement for movement of the chromatin fibre raises the possibility that chromatin loops continually escape from the surface of structural foci in order to probe the inter-chromatin space where favourable synthetic environments might be encountered. During this process, extended chromatin loops from neighboring territories might occupy the same nuclear space and so have a high probability of interacting, for example by binding to a common transcription factory. The analysis presented here suggests that at any time the extended loops represent a very small amount – ∼1% or less - of the mammalian genome. Even so, it is important to recognise that the single cell analysis used is unable to explore the range (spread) of isolated chromatin fibres and it remains an open question if extended chromatin fibres are able to persist as a result of stable interactions within the inter-chromatin space [1]–[3].

Our experiments imply that extended loops that spread from the surface of CTs are generally short-range and probably short-lived. If long-range (>µm) open loops are able to form in our experimental system, these must be rare in individual cells or below the level of detection of our analysis. In fact, chromatin loops that extend well outside the normal boundaries of CTs are not uncommon and provide an obvious means of increasing the range of inter-chromosomal contacts while maintaining the normal higher-order packaging density of DNA [36]. While such extruded loops provide one class of CT remodelling, we believe that our observations support a model in which the majority of inter-chromosomal contacts form locally at the boundaries of CTs, in domains where chromatin architecture might be open and dynamic. In this regard, it is notable that recent studies using electron spectroscopic imaging [47] have suggested that the majority of chromatin in mammalian cells is in the form of 10 nm chromatin fibers, which in differentiated cells fold locally to form higher-order DNA foci [30]. Interestingly, the chromatin fibers within embryonic stems cells appear to be much more chaotic, perhaps implying that ES and differentiated somatic cells have quite different principles of higher-order chromatin organization.

Analysis of widespread chromatin dynamics in different cell types supports this possibility. It is well-known that in differentiated cells chromatin structure is spatially stable over long periods of time [48] whereas similar experiments performed in ES cells shows their genome organization to be extremely plastic [Thomas Cremer, personal communication]. This implies that stable higher-order structures seen following differentiation are not a major chromatin feature in developmentally primitive cells. Even so, indirect functional evidence does show that some level of higher-order structure is present in stem cells. Notably, replication timing domains that are seen following cell commitment correlate with ∼0.5–1 Mbp chromatin domains – the DNA foci [8] – and similar replication structures are seen both in mouse ES cells and in their committed and differentiated descendants [42].

### Conclusions and perspectives

Genome-wide studies offer a promiscuous view of inter-chromosomal interactions, which suggest a significant degree of intermingling between DNA from different CTs (e.g. [41]). However, to date such experiments have been performed on large cell populations and provide a view of potential interactions without having the power to predict the frequency of these interactions within individual living cells. Moreover, unbiased analysis of potential genome interactions using Hi-C clearly shows that intra-chromosomal interactions within CTs are at least 2 orders of magnitude more frequent than inter-chromosomal interactions (see Figure 2 in [41]); this level is consistent with the potential for chromatin mixing described herein. Hence, while the formation of extensive interaction networks within mammalian cells appears to conflict with the idea that individual CTs are spatially self-contained [4], dynamic changes at the interaction interfaces of neighboring CTs (Fig. 5) can be sufficient to allow the formation of widespread gene interactions while preserving CTs as higher-order chromatin structures. As a growing body of evidence supports the formation of cell type specific ‘interactomes’ during cell differentiation [18]–[23], it is important to understand how different patterns of gene expression correlate with the formation of interaction networks and how these interactions define spatial and temporal changes in genome structure and function within individual cells.

## Materials and Methods

### Visualizing replication foci in human cells

HeLa cells were grown in the presence of different dTTP analogues to label sites of DNA synthesis, as described in detail by Maya-Mendoza et al. [49]. The following precursors were used: AlexaFluor488-dUTP (AF488-dUTP); Cy3-dUTP; biotin-dUTP and bromo-deoxyuridine (BrdU). AF488-dUTP and Cy3-dUTP were visualized either in living cells using time-lapse light microscopy or by confocal microscopy after fixation using routine procedures. For fixation, cells growing on glass coverslips were rinsed briefly in PBS (1 sec; 20°C to remove medium and fixed in 4% paraformaldehyde (15 min; 0°C). These fixation conditions preserved the structure of chromatin domains present in living cells and no changes in structure of the chromatin foci was seen under the imaging conditions used. Fixed cells were washed 3× in PBS, treated with 0.5% Triton ×100 in PBS, rinsed 3× in PBS, incubated with 5 µg/ml Hoechst 33258 (Sigma) for 10 min, rinsed 3× in PBS and mounted with either Vectashield or Prolong mounting media. Alternatively, DNA foci were labeled by indirect immuno-fluorescence [49]. Where secondary fluorescent antibodies were replaced by Qdots the following changes were applied: 1) permeabilization was altered to 1% Triton ×100 for 10 min; 2) Qdots (1/500 dilution) were applied to coverslips in 24 well plates and incubation performed for 15 h at 4°C with shaking (orbital rocker); fixation, primary antibody incubation and washes were as for routine immuno-labeling. We note that in our hands the performance of Qdots was very variable from batch to batch, with some batches giving high background staining. Qdots were from Invitrogen: streptavidin conjugated Qdot-525 was used to detect biotin labeled CTs and secondary anti-rat antibody conjugated with Qdot-605 to detect BrdU. TSA was purchased from Sigma. Western blotting was performed as described [50] using appropriate antibodies (Abcam), as shown.

For confocal imaging, samples were examined using a Zeiss LSM510META confocal microscope following well-established imaging protocols [27], [28]. Labeling conditions were selected to minimize background noise and the microscope configuration was selected to reduce bleed-through between imaging channels to negligible levels. In order to ensure optimal imaging performance, instrument alignment was performed at regular intervals by Zeiss. Multi-coloured TetraSpeck florescent beads were used to monitor point spread functions and correct chromatic shift; maximum tolerated shifts were 50 nm in X-Y and 100 nm in Z. To minimise chromatic aberrations, great care was also taken to balance labeling intensities in different imaging channels. Confocal sections were collected through a 100× (1.45 NA) lens and 3-D images generated using Z stacks and processed in Imaris® software. For LSM510 image acquisition the following channel settings were used: green −488 nm laser line at 2% intensity with a BP 500–530 IR filter; red – 543 nm laser line at 32% of intensity and LP 545 filter. 4-D time-lapse imaging was performed using either a DeltaVision microscope with a CoolSNAP-HQ2 camera and Olympus objective (100×; 1.4 NA) or Zeiss LSM510META confocal microscope using the settings detailed above. The Deltavision system was used for long-term imaging experiments (e.g. Fig. 1), with the intensity of light during imaging kept to 32% using an acquisition speed of 100–200 ms. The conditions used allow imaging for at least 2 days without influencing cell viability or cell cycle parameters. Because of the zoom facilities, the Zeiss system was used when foci-level resolution was required (Fig. 5). As above, the light intensity was reduced to the minimum required to resolve individual foci and the imaging conditions used were shown not to prevent subsequent cell division.

For detailed co-localization analysis (Fig. 2), confocal imaging was performed using a Zeiss LSM710 microscope using instrument setting equivalent to those detailed above to minimize bleed-through between channels and background levels. Z-stacks were acquired for each sample with voxel dimensions of 0.8×0.8×0.34 microns, for X, Y and Z respectively with an XY resolution of 988×988 pixels and a pinhole setting of 1.0 Airy unit. Amplifier and detector gain and offset were optimally chosen by the instrument for each field acquired. For the Alexa-488 channel an EF1 filter set was used with a SPI wavelength range from 493–543 nm. For the Cy3 channel an EF2 filter set was used with a SPI wavelength range from 566–681 nm.

### Image analysis and model building

3-D and 4-D images were analyzed using Imaris® software (Bitplane). For confocal images, Z stacks were processed using Imaris® software after applying a Gaussian or Median filter. Imaris® software was used to process 3-D projections, identify individual foci and assign coordinates for mass centers of each focus. Individual channels were processed separately. Co-ordinates of mass centers were used to define the spatial relationship between adjacent foci, either within or between channels. The mass centers can be represented by computer generated spheres that correspond in size to average foci. Such images are artificial and while providing an accurate representation of the positions of foci are not intended to provide a realistic representation of the foci themselves. Imaris® imaging software was used to isolate cropped regions, with the same crop volumes used for equivalent samples.

For high-throughput image analysis, in-house scripts were developed using Fiji software [51] with the aid of the suite of 3-D filters [52]. Co-localization analysis was performed with JACoP [28] and co-localized volumes estimated by multiplying the number of co-localized voxels by the volume covered by a single voxel. Co-localized voxels where defined as voxels for which both channels indicated values above a threshold point, equal to the standard deviation of the distribution of pixel intensities in the corresponding channel.

To visualize 3-D interactions between CTs (e.g. Fig. 2D), coordinates of each of the fluorescent tags were exported individually into Virtual Reality Modelling Language (VRML) format using Imaris® software. VRML files were exported to 3ds format using an open-source, platform-free 3d-design suite (http://www.blender.org/). These files were imported into Autodesk® 3ds Max® (www.autodesk.com/3dsmax) and imported files merged in a single MAX file to facilitate image rendering, 3-D modelling and animation. This procedure using 3ds built-in compound modifiers models the 3-D shape of the chromatin compartment using the continuity of labeled DNA foci to define the chromatin space.

## Supporting Information

Figure S1 **Analysis of co-localization in cells labeled with 488-dUTP and Cy3-dUTP.** This figure demonstrates how manual thresholding was used for quantitative channel co-localization. The sample described in Figure 1 was used for analysis and the specific nucleus shown in (Cii) use in this example. As in Figure 1, LSM (Zeiss) data files were uploaded into Imaris software and individual channels (3-D) isolated (A: three images on top show the unprocessed green (left) and red (center) channels and the channel merge (right; co-localized sites are shown yellow). Data from the imaging files is extracted as a screen shot on the right. Using data like this we performed a detailed empirical analysis of the behavior of sites of co-localization, using co-localization intensity plots (B). Using these plots, manual thresholding was applied to eliminate background noise. Threshold settings (shown by yellow lines in the intensity co-loclization plot (B)) were adjusted sequentially in order to establish the minimum level that eliminated noise that was clearly unrelated to the real signal (define by nuclear location). Using this approach, the minimum value for thresholding correlated with the standard deviation of the data intensity in the separate imaging channels. When voxels below this intensity were subtracted from the images noise was essentially eliminated without degrading the structure of the true signal (in A, compare raw images (top panel) and equivalent images after noise reduction (lower panel)). Following nosie reduction, the filtered images were analyzed to identify levels of co-localization (data panel below). Finally, the voxels showing co-localization after background subtraction were extracted (C), for comparison with levels of apparent co-localization in the primary image (A: yellow voxels, top right).
(TIF)

Video S1 **Preservation of relative spatial architecture of CTs in response to cell movement.** Video showing the time-lapse series that includes the individual images shown in Figure 1B. Video rate - 1 frame/sec. 0 to 360 mins.
(MOV)

Video S2 **Regions of apparent co-localization between neighboring CTs result from foci that lie in close juxtaposition in nuclear space.** This video shows how co-localization alters during Z sectioning of the space-filling model presented in Figure 1Di. Note that while zones of apparent co-localization often appear along the borders where neighboring CTs meet (these appear yellow while panning through the image) high-resolution analysis shows that these rarely represent true co-localization. In fact, sectioning through the nucleus shows almost complete separation of the green- and red-labeled DNA.
(AVI)

Video S3 **CT architecture generates frequent regions of interdigitation along the boundaries where neighboring CTs meet.** This video shows a high-magnification 3D rotational view of the region shown in Figure 1Dii–iii(from the region highlighted (white box) in Fig. 1Di). Note that domains protruding from the surface of both CTs are able to pass into the neighboring territory. However, while foci from the individual CTs interact within the same nuclear space the structural integrity of the foci appears to be preserved so that DNA interactions are restricted to the surfaces where adjacent foci touch. Such experiments do not support the existence of extensive nuclear domains where DNA from two or more CTs is freely mixed, although it is important to note that DNA within individual foci will also be dynamic so that DNA at the surface of individual DNA foci will also change with time.
(AVI)
