# Supplementary figures and images for: Innate Structure of DNA Foci Restricts the Mixing of DNA from Different Chromosome Territories
Source: PLoS One. 2011 Dec 21;6(12):e27527. doi: 10.1371/journal.pone.0027527 (PMC3244381; doi:10.1371/journal.pone.0027527)

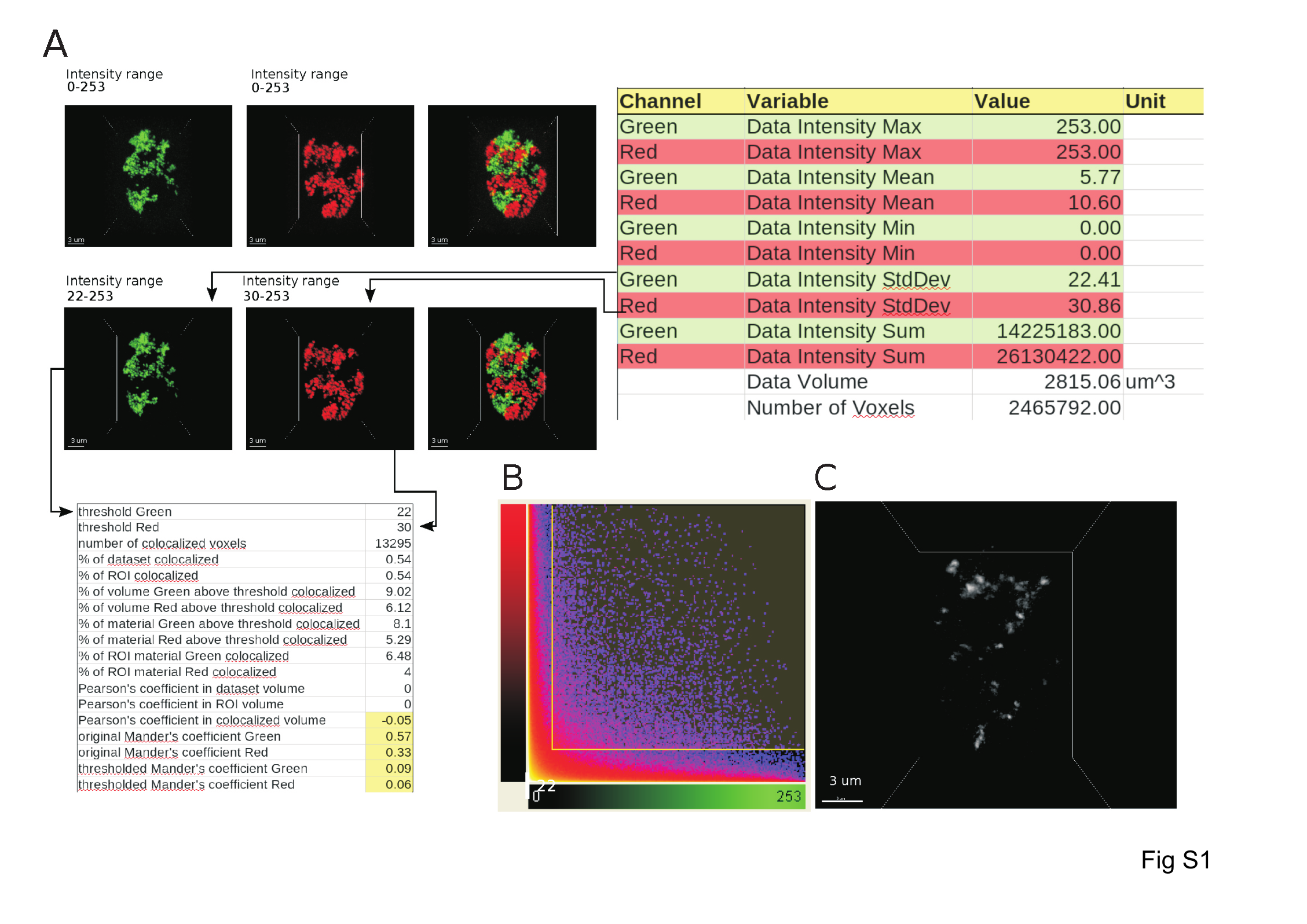

Supplement: Figure S1 — Analysis of co-localization in cells labeled with 488-dUTP and Cy3-dUTP. This figure demonstrates how manual thresholding was used for quantitative channel co-localization. The sample described in Figure 1 was used for analysis and the specific nucleus shown in (Cii) use in this example. As in Figure 1, LSM (Zeiss) data files were uploaded into Imaris software and individual channels (3-D) isolated (A: three images on top show the unprocessed green (left) and red (center) channels and the channel merge (right; co-localized sites are shown yellow). Data from the imaging files is extracted as a screen shot on the right. Using data like this we performed a detailed empirical analysis of the behavior of sites of co-localization, using co-localization intensity plots (B). Using these plots, manual thresholding was applied to eliminate background noise. Threshold settings (shown by yellow lines in the intensity co-loclization plot (B)) were adjusted sequentially in order to establish the minimum level that eliminated noise that was clearly unrelated to the real signal (define by nuclear location). Using this approach, the minimum value for thresholding correlated with the standard deviation of the data intensity in the separate imaging channels. When voxels below this intensity were subtracted from the images noise was essentially eliminated without degrading the structure of the true signal (in A, compare raw images (top panel) and equivalent images after noise reduction (lower panel)). Following nosie reduction, the filtered images were analyzed to identify levels of co-localization (data panel below). Finally, the voxels showing co-localization after background subtraction were extracted (C), for comparison with levels of apparent co-localization in the primary image (A: yellow voxels, top right). (TIF) [file pone.0027527.s001.tif]
